# Genistein-3′-sulfonic acid dihydrate

**DOI:** 10.1107/S1600536809014767

**Published:** 2009-04-30

**Authors:** Chang-Peng Zhang, Hao Ni, Hai-Yan Tu, Yong-Rong Xie, Rui-Qing Yang

**Affiliations:** a Key Laboratory of Jiangxi University for Functional Materials Chemistry, Department of Chemistry and Life Science, Gannan Normal University, Ganzhou, Jiangxi 341000, People’s Republic of China

## Abstract

In the title compound [systematic name: 5-(5,7-dihydr­oxy-4-oxo-4*H*-chromen­yl)-2-hydroxy­benzene­sulfonic acid dihydrate], C_15_H_10_O_8_S·2H_2_O, the benzopyran­one ring is not coplanar with the phenyl ring, the dihedral angle between them being 41.35 (3)°. No H atom was placed on the sulphonic acid group because it was not possible to distinguish between the two S=O bonds and the S—O bond. In the crystal, the mol­ecules are linked by classical O—H⋯O and C—H⋯O intra- and inter­molecular hydrogen bonds and aromatic π–π stacking inter­actions [centroid–centroid distance of 3.4523 (14) Å between the 1, 4-pyran­one rings and the benzene rings, and 3.6337 (14) Å between the benzene rings] into a supra­molecular structure.

## Related literature

Genistein is an isoflavone that can be extracted from plants such as soybean, trifolium, puerarin, see: Curnow *et al.* (1955 ▶); Kaufman *et al.* (1997 ▶). For its anti-tumour, anti-arteriosclerosis and anti-bone loss properties, see: Fritz *et al.* (1998 ▶); Zhu *et al.* (2006 ▶). It can also reduce plasma lipids and kill various cancer cells without damaging normal cells, see: Fanti *et al.* (1998 ▶); Lamartiniere (2000 ▶). It has poor solubility in water and fat (Suo *et al.*, 2005 ▶). One effective way to increase the solubility of these compounds is to involve a sulfonate group, see: Kopacz (1981 ▶); Pusz *et al.* (2001 ▶); Xie *et al.* (2002 ▶).
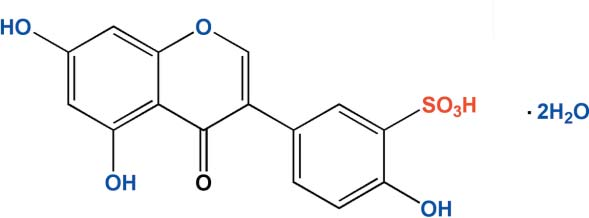


## Experimental

### 

#### Crystal data

C_15_H_10_O_8_S·2H_2_O
*M* *_r_* = 386.31
Triclinic, 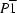

*a* = 7.9100 (4) Å
*b* = 8.1977 (3) Å
*c* = 14.3431 (7) Å
α = 73.626 (3)°
β = 80.346 (3)°
γ = 65.498 (3)°
*V* = 810.61 (6) Å^3^
*Z* = 2
Mo *K*α radiation
μ = 0.26 mm^−1^
*T* = 296 K
0.20 × 0.20 × 0.05 mm

#### Data collection

Bruker SMART APEXII CCD area-detector diffractometer
Absorption correction: multi-scan (*SADABS*; Sheldrick, 1996 ▶) *T*_min_ = 0.952, *T*_max_ = 0.988
7136 measured reflections
3736 independent reflections
2466 reflections with *I* > 2σ(*I*)
*R*_int_ = 0.037

#### Refinement

*R*[*F* ^2^ > 2σ(*F* ^2^)] = 0.047
*wR*(*F* ^2^) = 0.114
*S* = 0.95
3736 reflections
262 parameters
H-atom parameters constrained
Δρ_max_ = 0.52 e Å^−3^
Δρ_min_ = −0.47 e Å^−3^

### 

Data collection: *SMART* (Bruker, 2004 ▶); cell refinement: *SAINT* (Bruker, 2004 ▶); data reduction: *SAINT* (Bruker, 2004 ▶); program(s) used to solve structure: *SHELXS97* (Sheldrick, 2008 ▶); program(s) used to refine structure: *SHELXL97* (Sheldrick, 2008 ▶); molecular graphics: *SHELXTL* (Sheldrick, 2008 ▶); software used to prepare material for publication: *SHELXTL* (Sheldrick, 2008 ▶).

## Supplementary Material

Crystal structure: contains datablocks I, global. DOI: 10.1107/S1600536809014767/fl2244sup1.cif

Structure factors: contains datablocks I. DOI: 10.1107/S1600536809014767/fl2244Isup2.hkl

Additional supplementary materials:  crystallographic information; 3D view; checkCIF report

## Figures and Tables

**Table 1:** Hydrogen-bond geometry (Å, °)

*D*—H⋯*A*	*D*—H	H⋯*A*	*D*⋯*A*	*D*—H⋯*A*
O2*W*—H1⋯O1*W*	0.85	2.07	2.915 (3)	173
O2*W*—H2⋯O7*A*^i^	0.85	2.17	2.999 (15)	165
O2*W*—H2⋯O6*A*^ii^	0.85	2.58	2.903 (13)	104
O2—H2*A*⋯O1	0.82	1.85	2.580 (2)	148
O1*W*—H3⋯O8*A*^iii^	0.85	2.23	2.968 (7)	146
O3—H3*A*⋯O8*A*^iv^	0.82	1.89	2.705 (8)	171
O1*W*—H4⋯O5	0.85	2.18	3.000 (2)	162
O5—H5*A*⋯O6*A*	0.82	2.40	2.835 (12)	114
O5—H5*A*⋯O6*A*^v^	0.82	2.05	2.784 (14)	148
C6—H6*A*⋯O7*A*^vi^	0.93	2.44	3.356 (15)	169
C8—H8*A*⋯O2^iii^	0.93	2.31	3.217 (3)	164

Symmetry codes: (i) 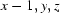; (ii) 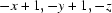; (iii) 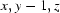; (iv) 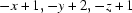; (v) 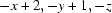; (vi) 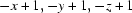.

**Fig. 1.**
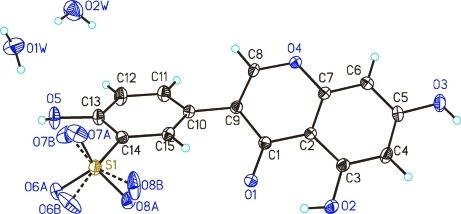
The asymmetric unit of (I), with displacement ellipsoids drawn at the 30% probability level.

**Fig. 2.**
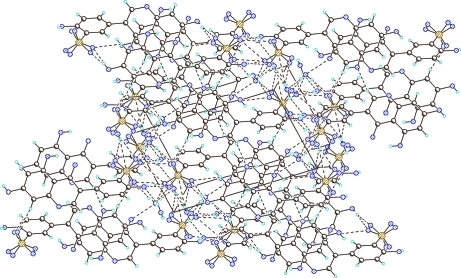
The hydrogen-bonding motif in (I). Dashed lines indicate the hydrogen bonds.

**Fig. 3.**
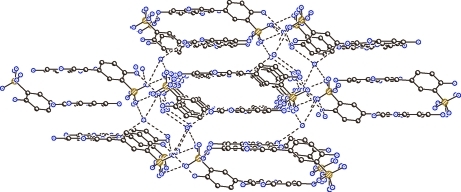
View of the π-π stacking for compound (I) along the b axis.

## supplementary crystallographic information

### Comment

Genistein is an isoflavone that can be extracted from plants such as soybean, trifolium, puerarin (Curnow *et al.* 1955; Kaufman *et al.*, 1997). It has physiological functions of anti-tumour, anti-arteriosclerosis, anti-bone loss (Fritz *et al.*, 1998; Zhu *et al.*, 2006). It can also reduce plasma lipids and kill various cancer cells without damaging normal cells (Fanti *et al.*, 1998; Lamartiniere, 2000). Nevertheless, its medical applications are restricted because of its poor solubility in water and fat (Suo *et al.*,2005). One effective way to increase the solubility of these compounds is to involve a sulfonate group (Kopacz, 1981; Pusz *et al.*, 2001; Xie *et al.* 2002).

We present here the structure of (I, Fig. 1), a new derivative of Genistein. In (I) the molecules are linked by classic O—H···O and C—H···O intra- and intermolecular hydrogen bonds (Table 1). Adjacent benzopyranone rings are aligned in a parallel and alternatively inverse fashion, with a centroid-centroid distance of 3.4523 (14)Å between 1, 4-pyranone rings and benzene rings, and 3.6337 (14)Å between the benzene rings (Table 2), indicating significant stacking interactions that form columns running along the *a* axis. The hydrogen bonding and π-π stacking interactions extend the structure into a 3-D supramolecular structure (Fig. 2 and Fig. 3).

### Experimental

In a 100 ml flask are placed 40 ml 98% sulfuric acid and 10 g (37 mmol) genistein with stirrer. The resulting mixture is stirred at room temperature for 6 h. The reaction mixture is carefully diluted by addition of 40 ml ice water. The resulting yellow solid is filtered, and recrystallized from 50 ml of 90% acetonitrile to give 9.1–11 g (70–85%) of the title compound yellow crystals.

### Refinement

The analysis indicated that all three O atoms of the sulfonate group are disordered and therefore the refinement did not converge satisfactorily. Two sulfonate groups, with an occupancies of 0.53740 (O6A, O7A, O8A) and 0.46260(O6B, O7B, O8B), respectively, could be detected and refined. No H atom was added to the sulfonate group because it was not possible to distinguish between the 2 S=O bonds and the S—O bond. The disorder in the SO_3_ group was treated with the tools available in *SHELXL97* (Sheldrick, 2008).

H atoms bonded to C atoms were placed in calculated positions, with C—H = 0.93 Å and *U*_iso_(H) = 1.2Ueq(C), and were included in the refinement in the riding-model approximation. The H atoms of water molecules were located in difference Fourier maps and then idealized and treated as riding, with O—H = 0.82–0.85 Å and *U*_iso_(H) = 1.2Ueq(O).

### Crystal data

**Table:**

C_15_H_10_O_8_S·2H_2_O	*V* = 810.61 (6) Å^3^
*M**_r_* = 386.31	*Z* = 2
Triclinic, *P*1	*F*(000) = 398
Hall symbol: -P 1	*D*_x_ = 1.579 Mg m^−^^3^
*a* = 7.9100 (4) Å	Mo *K*α radiation, λ = 0.71073 Å
*b* = 8.1977 (3) Å	θ = 2.8–27.6°
*c* = 14.3431 (7) Å	µ = 0.26 mm^−^^1^
α = 73.626 (3)°	*T* = 296 K
β = 80.346 (3)°	Block, yellow
γ = 65.498 (3)°	0.20 × 0.20 × 0.05 mm

### Data collection

**Table:**

Bruker SMART APEXII CCD area-detector diffractometer	3736 independent reflections
Radiation source: fine-focus sealed tube	2466 reflections with *I* > 2σ(*I*)
graphite	*R*_int_ = 0.037
φ and ω scans	θ_max_ = 27.6°, θ_min_ = 2.8°
Absorption correction: multi-scan (*SADABS*; Sheldrick, 1996)	*h* = −9→10
*T*_min_ = 0.952, *T*_max_ = 0.988	*k* = −10→10
7136 measured reflections	*l* = −16→18

### Refinement

**Table:**

Refinement on *F*^2^	Primary atom site location: structure-invariant direct methods
Least-squares matrix: full	Secondary atom site location: difference Fourier map
*R*[*F*^2^ > 2σ(*F*^2^)] = 0.047	Hydrogen site location: inferred from neighbouring sites
*wR*(*F*^2^) = 0.114	H-atom parameters constrained
*S* = 0.95	*w* = 1/[σ^2^(*F*_o_^2^) + (0.0499*P*)^2^] where *P* = (*F*_o_^2^ + 2*F*_c_^2^)/3
3736 reflections	(Δ/σ)_max_ < 0.001
262 parameters	Δρ_max_ = 0.52 e Å^−^^3^
0 restraints	Δρ_min_ = −0.47 e Å^−^^3^

### Special details

**Table:**

Geometry. All e.s.d.'s (except the e.s.d. in the dihedral angle between two l.s. planes) are estimated using the full covariance matrix. The cell e.s.d.'s are taken into account individually in the estimation of e.s.d.'s in distances, angles and torsion angles; correlations between e.s.d.'s in cell parameters are only used when they are defined by crystal symmetry. An approximate (isotropic) treatment of cell e.s.d.'s is used for estimating e.s.d.'s involving l.s. planes.
Refinement. Refinement of *F*^2^ against ALL reflections. The weighted *R*-factor *wR* and goodness of fit *S* are based on *F*^2^, conventional *R*-factors *R* are based on *F*, with *F* set to zero for negative *F*^2^. The threshold expression of *F*^2^ > σ(*F*^2^) is used only for calculating *R*-factors(gt) *etc*. and is not relevant to the choice of reflections for refinement. *R*-factors based on *F*^2^ are statistically about twice as large as those based on *F*, and *R*- factors based on ALL data will be even larger.

### Fractional atomic coordinates and isotropic or equivalent isotropic displacement parameters (Å^2^)

**Table:**

	*x*	*y*	*z*	*U*_iso_*/*U*_eq_	Occ. (<1)
S1	0.91573 (8)	0.62831 (8)	0.13396 (4)	0.02871 (17)	
O1	0.3853 (2)	0.9816 (2)	0.33901 (11)	0.0349 (4)	
O2	0.2966 (2)	1.1865 (2)	0.46121 (12)	0.0463 (5)	
H2A	0.3371	1.1535	0.4101	0.056*	
O3	0.0060 (2)	0.9940 (2)	0.76568 (11)	0.0441 (5)	
H3A	0.0326	1.0699	0.7804	0.053*	
O4	0.1972 (2)	0.62379 (19)	0.54241 (11)	0.0325 (4)	
O5	0.7199 (2)	0.4397 (2)	0.05141 (12)	0.0483 (5)	
H5A	0.8324	0.4128	0.0492	0.058*	
C1	0.3351 (3)	0.8670 (3)	0.40347 (15)	0.0263 (5)	
C2	0.2545 (3)	0.9026 (3)	0.49667 (15)	0.0246 (5)	
C3	0.2355 (3)	1.0616 (3)	0.52404 (16)	0.0296 (5)	
C4	0.1563 (3)	1.0929 (3)	0.61293 (17)	0.0339 (6)	
H4A	0.1465	1.1976	0.6303	0.041*	
C5	0.0900 (3)	0.9663 (3)	0.67758 (16)	0.0305 (5)	
C6	0.1018 (3)	0.8113 (3)	0.65325 (16)	0.0299 (5)	
H6A	0.0550	0.7290	0.6959	0.036*	
C7	0.1848 (3)	0.7812 (3)	0.56408 (16)	0.0263 (5)	
C8	0.2829 (3)	0.5863 (3)	0.45676 (16)	0.0313 (5)	
H8A	0.2944	0.4762	0.4448	0.038*	
C9	0.3528 (3)	0.6939 (3)	0.38735 (15)	0.0261 (5)	
C10	0.4469 (3)	0.6336 (3)	0.29699 (15)	0.0265 (5)	
C11	0.3792 (3)	0.5469 (3)	0.25092 (17)	0.0352 (6)	
H11A	0.2693	0.5304	0.2753	0.042*	
C12	0.4730 (3)	0.4846 (3)	0.16923 (18)	0.0392 (6)	
H12A	0.4249	0.4277	0.1391	0.047*	
C13	0.6385 (3)	0.5062 (3)	0.13165 (16)	0.0317 (5)	
C14	0.7058 (3)	0.5965 (3)	0.17563 (15)	0.0250 (5)	
C15	0.6094 (3)	0.6585 (3)	0.25784 (15)	0.0259 (5)	
H15A	0.6554	0.7181	0.2872	0.031*	
O1W	0.6658 (3)	0.1139 (2)	0.02597 (15)	0.0646 (6)	
H3	0.7677	0.0288	0.0470	0.078*	
H4	0.6800	0.2140	0.0191	0.078*	
O2W	0.3261 (3)	0.0875 (3)	0.13170 (15)	0.0775 (7)	
H1	0.4261	0.0988	0.1045	0.093*	
H2	0.2363	0.1863	0.1398	0.093*	
O6A	0.9142 (16)	0.6736 (12)	0.0261 (8)	0.0346 (14)	0.54
O7A	1.067 (2)	0.456 (2)	0.1681 (9)	0.043 (2)	0.54
O8A	0.8999 (11)	0.7821 (10)	0.1651 (4)	0.0369 (13)	0.54
O6B	0.899 (2)	0.7323 (15)	0.0381 (10)	0.067 (3)	0.46
O7B	1.059 (2)	0.450 (3)	0.1480 (12)	0.069 (5)	0.46
O8B	0.9427 (13)	0.7255 (13)	0.2012 (5)	0.061 (3)	0.46

### Atomic displacement parameters (Å^2^)

**Table:**

	*U*^11^	*U*^22^	*U*^33^	*U*^12^	*U*^13^	*U*^23^
S1	0.0283 (3)	0.0327 (3)	0.0301 (3)	−0.0149 (3)	0.0043 (2)	−0.0138 (3)
O1	0.0453 (10)	0.0311 (9)	0.0315 (9)	−0.0208 (8)	0.0095 (7)	−0.0101 (7)
O2	0.0694 (13)	0.0418 (10)	0.0434 (11)	−0.0385 (10)	0.0200 (9)	−0.0212 (8)
O3	0.0580 (12)	0.0550 (11)	0.0329 (10)	−0.0313 (10)	0.0116 (8)	−0.0246 (8)
O4	0.0455 (10)	0.0261 (8)	0.0295 (9)	−0.0185 (8)	0.0071 (7)	−0.0101 (7)
O5	0.0406 (10)	0.0809 (13)	0.0468 (11)	−0.0340 (10)	0.0169 (8)	−0.0454 (10)
C1	0.0241 (11)	0.0258 (11)	0.0273 (12)	−0.0086 (10)	−0.0008 (9)	−0.0061 (10)
C2	0.0267 (11)	0.0224 (11)	0.0249 (12)	−0.0098 (9)	0.0003 (9)	−0.0068 (9)
C3	0.0311 (12)	0.0291 (12)	0.0326 (13)	−0.0152 (10)	0.0024 (10)	−0.0105 (10)
C4	0.0380 (13)	0.0367 (13)	0.0367 (14)	−0.0181 (11)	0.0011 (10)	−0.0197 (11)
C5	0.0292 (12)	0.0394 (13)	0.0235 (12)	−0.0107 (11)	0.0007 (9)	−0.0133 (10)
C6	0.0332 (13)	0.0296 (12)	0.0269 (12)	−0.0140 (10)	0.0017 (9)	−0.0056 (10)
C7	0.0267 (12)	0.0236 (11)	0.0287 (12)	−0.0085 (10)	−0.0020 (9)	−0.0080 (10)
C8	0.0374 (13)	0.0267 (12)	0.0318 (13)	−0.0123 (11)	0.0066 (10)	−0.0154 (10)
C9	0.0250 (11)	0.0253 (11)	0.0280 (12)	−0.0090 (10)	0.0009 (9)	−0.0091 (10)
C10	0.0275 (11)	0.0263 (11)	0.0253 (12)	−0.0090 (10)	0.0012 (9)	−0.0093 (9)
C11	0.0296 (13)	0.0463 (14)	0.0379 (14)	−0.0203 (12)	0.0054 (10)	−0.0180 (12)
C12	0.0363 (14)	0.0562 (16)	0.0432 (15)	−0.0268 (13)	0.0056 (11)	−0.0299 (13)
C13	0.0318 (13)	0.0403 (13)	0.0272 (12)	−0.0145 (11)	0.0030 (9)	−0.0163 (11)
C14	0.0239 (11)	0.0253 (11)	0.0257 (12)	−0.0096 (9)	−0.0003 (9)	−0.0064 (9)
C15	0.0257 (11)	0.0262 (11)	0.0272 (12)	−0.0084 (10)	−0.0033 (9)	−0.0101 (9)
O1W	0.0601 (13)	0.0449 (11)	0.0873 (16)	−0.0203 (10)	−0.0079 (11)	−0.0116 (11)
O2W	0.0639 (15)	0.0834 (15)	0.0737 (16)	−0.0237 (13)	0.0042 (11)	−0.0139 (12)
O6A	0.036 (2)	0.045 (3)	0.023 (2)	−0.015 (3)	0.0032 (16)	−0.011 (2)
O7A	0.029 (3)	0.051 (6)	0.043 (3)	−0.018 (3)	−0.009 (2)	0.005 (3)
O8A	0.048 (3)	0.048 (3)	0.035 (3)	−0.031 (2)	0.003 (2)	−0.024 (2)
O6B	0.056 (5)	0.095 (9)	0.041 (6)	−0.041 (6)	0.002 (4)	0.014 (5)
O7B	0.025 (5)	0.032 (5)	0.129 (13)	0.000 (4)	0.019 (6)	−0.018 (7)
O8B	0.051 (5)	0.123 (8)	0.053 (5)	−0.058 (5)	0.022 (3)	−0.065 (5)

### Geometric parameters (Å, °)

**Table:**

S1—O6B	1.394 (14)	C4—C5	1.399 (3)
S1—O8A	1.404 (7)	C4—H4A	0.9300
S1—O7B	1.412 (16)	C5—C6	1.374 (3)
S1—O7A	1.438 (14)	C6—C7	1.375 (3)
S1—O6A	1.487 (11)	C6—H6A	0.9300
S1—O8B	1.501 (8)	C8—C9	1.342 (3)
S1—C14	1.766 (2)	C8—H8A	0.9300
O1—C1	1.262 (2)	C9—C10	1.487 (3)
O2—C3	1.356 (3)	C10—C15	1.385 (3)
O2—H2A	0.8207	C10—C11	1.389 (3)
O3—C5	1.358 (2)	C11—C12	1.384 (3)
O3—H3A	0.8205	C11—H11A	0.9300
O4—C8	1.350 (2)	C12—C13	1.392 (3)
O4—C7	1.372 (2)	C12—H12A	0.9300
O5—C13	1.360 (2)	C13—C14	1.390 (3)
O5—H5A	0.8206	C14—C15	1.395 (3)
C1—C2	1.435 (3)	C15—H15A	0.9300
C1—C9	1.447 (3)	O1W—H3	0.8508
C2—C7	1.401 (3)	O1W—H4	0.8502
C2—C3	1.408 (3)	O2W—H1	0.8500
C3—C4	1.366 (3)	O2W—H2	0.8500
			
O8A—S1—O7A	117.5 (7)	C6—C5—C4	121.3 (2)
O8A—S1—O6A	110.1 (4)	C5—C6—C7	118.1 (2)
O7A—S1—O6A	110.5 (6)	C5—C6—H6A	121.0
O8A—S1—C14	105.6 (3)	C7—C6—H6A	121.0
O7A—S1—C14	107.8 (7)	O4—C7—C6	116.80 (19)
O6A—S1—C14	104.3 (5)	O4—C7—C2	120.09 (19)
O6B—S1—O7B	115.3 (8)	C6—C7—C2	123.1 (2)
O6B—S1—O8B	112.3 (5)	C9—C8—O4	125.7 (2)
O7B—S1—O8B	107.7 (8)	C9—C8—H8A	117.2
O6B—S1—C14	108.5 (6)	O4—C8—H8A	117.2
O8A—S1—C14	105.6 (3)	C8—C9—C1	118.4 (2)
O7B—S1—C14	106.3 (9)	C8—C9—C10	119.97 (19)
O7A—S1—C14	107.8 (7)	C1—C9—C10	121.67 (18)
O6A—S1—C14	104.3 (5)	C15—C10—C11	118.0 (2)
O8B—S1—C14	106.2 (4)	C15—C10—C9	120.2 (2)
C3—O2—H2A	109.4	C11—C10—C9	121.7 (2)
C5—O3—H3A	109.5	C12—C11—C10	120.9 (2)
C8—O4—C7	118.66 (16)	C12—C11—H11A	119.5
C13—O5—H5A	109.4	C10—C11—H11A	119.5
O1—C1—C2	121.64 (19)	C11—C12—C13	120.7 (2)
O1—C1—C9	122.13 (19)	C11—C12—H12A	119.6
C2—C1—C9	116.23 (18)	C13—C12—H12A	119.6
C7—C2—C3	116.69 (19)	O5—C13—C14	124.9 (2)
C7—C2—C1	120.75 (19)	O5—C13—C12	116.1 (2)
C3—C2—C1	122.53 (19)	C14—C13—C12	119.0 (2)
O2—C3—C4	119.2 (2)	C13—C14—C15	119.4 (2)
O2—C3—C2	119.6 (2)	C13—C14—S1	122.28 (16)
C4—C3—C2	121.2 (2)	C15—C14—S1	118.27 (17)
C3—C4—C5	119.6 (2)	C10—C15—C14	121.9 (2)
C3—C4—H4A	120.2	C10—C15—H15A	119.1
C5—C4—H4A	120.2	C14—C15—H15A	119.1
O3—C5—C6	117.1 (2)	H3—O1W—H4	105.1
O3—C5—C4	121.6 (2)	H1—O2W—H2	116.1

### Hydrogen-bond geometry (Å, °)

**Table:**

*D*—H···*A*	*D*—H	H···*A*	*D*···*A*	*D*—H···*A*
O2W—H1···O1W	0.85	2.07	2.915 (3)	173
O2W—H2···O7A^i^	0.85	2.17	2.999 (15)	165
O2W—H2···O6A^ii^	0.85	2.58	2.903 (13)	104
O2—H2A···O1	0.82	1.85	2.580 (2)	148
O1W—H3···O8A^iii^	0.85	2.23	2.968 (7)	146
O3—H3A···O8A^iv^	0.82	1.89	2.705 (8)	171
O1W—H4···O5	0.85	2.18	3.000 (2)	162
O5—H5A···O6A	0.82	2.40	2.835 (12)	114
O5—H5A···O6A^v^	0.82	2.05	2.784 (14)	148
C6—H6A···O7A^vi^	0.93	2.44	3.356 (15)	169
C8—H8A···O2^iii^	0.93	2.31	3.217 (3)	164
C15—H15A···O1	0.93	2.52	2.944 (3)	108
C15—H15A···O8A	0.93	2.50	2.868 (9)	104

Symmetry codes:  (i) *x*−1, *y*, *z*; (ii) −*x*+1, −*y*+1, −*z*; (iii) *x*, *y*−1, *z*; (iv) −*x*+1, −*y*+2, −*z*+1; (v) −*x*+2, −*y*+1, −*z*; (vi) −*x*+1, −*y*+1, −*z*+1.

### Table 2 π-π interactions (Å, °)

**Table:**

Cg	Cg	α	DCC	τ
Cg1	Cg2^i^	1.38	3.4524 (14)	15.63
Cg2	Cg2^i^	0.03	3.6336 (14)	24.54

Symmetry code: (i) -x, 2-y, 1-z. α is dihedral angle between the planes, DCC is the length of the CC vector (centroid–centroid), τ is the angle(s) subtended by the plane normal(s) to CC. Cg1 is the centroid of ring O4, C1, C2, C7–C9 and Cg2 is the centroid of ring C2–C7.
